# Molecular Identification of Multidrug-Resistant *Campylobacter* Species From Diarrheal Patients and Poultry Meat in Shanghai, China

**DOI:** 10.3389/fmicb.2018.01642

**Published:** 2018-07-31

**Authors:** Yinju Du, Chuanqing Wang, Yulong Ye, Yue Liu, Aimin Wang, Yong Li, Xiaoying Zhou, Hanjian Pan, Jianmin Zhang, Xuebin Xu

**Affiliations:** ^1^Department of Microbiology, Center for Disease Control and Prevention of Liaocheng City, Shandong, China; ^2^Department of Microbiology, Children’s Hospital of Fudan University, Shanghai, China; ^3^Department of Microbiology, The Jinshan District Center for Disease Control and Prevention, Shanghai, China; ^4^Department of Food Microbiology, Shanghai Municipal Center for Disease Control and Prevention, Shanghai, China; ^5^Department of Food Microbiology, The Putuo District Center for Disease Control and Prevention, Shanghai, China; ^6^Shanghai Municipal Di-Jing Technology Center for Microbiology, Shanghai, China; ^7^Department of Food Science and Technology, School of Agriculture and Biology, Shanghai Jiao Tong University, Shanghai, China; ^8^Key Laboratory of Zoonosis Prevention and Control of Guangdong Province, College of Veterinary Medicine, South China Agricultural University, Guangzhou, China; ^9^Department of Microbiology, Shanghai Municipal Center for Disease Control and Prevention, Shanghai, China

**Keywords:** *Campylobacter*, PFGE, antimicrobial susceptibility, multidrug resistance, efflux pump

## Abstract

Emerging resistance to the antimicrobial agents of choice for treatment of thermophilic *Campylobacter* infections is becoming a serious threat to public health. In this study, 548 *Campylobacter* (372 *C. jejuni* and 176 *C. coli*) isolates from diarrheal patients and poultry meat were subjected for antibiotic susceptibility analysis to ciprofloxacin, tetracycline, gentamicin, erythromycin and clindamycin. Among them, 151 *Campylobacter* (32 *C. jejuni* and 119 *C. coli*) were identified as multidrug resistant isolates. PFGE analysis was performed on the 151 multidrug resistant isolates to determine their genetic relatedness, and 103 PFGE genotypes were determined. Some isolates from both human and chicken belonged to identical genotypes, indicating these clones might be able to spread between human and chicken. Antibiotic resistant genes of the 151 isolates were identified. The numbers of isolates carried *tet (O)*, *aadE, ermB*, and *aadE-sat4-aphA* were 148 (98%), 89 (58.9%), 31 (20.5%), and 10 (6.6%), respectively. Almost all (*n* = 150, 99.3%) had *gyrA* mutation at codon 86. And the 23s rRNA A2075G point mutation was found in 56 (37.1%) isolates. Gene mutations at the *cmeR-cmeABC* intergenic region may lead to the activation of CmeABC multidrug efflux pump, and in this study novel sequence types of the intergenic region were identified in both *C. jejuni* and *C. coli*. This study determined the genetic prerequisites for antibiotic resistance of multidrug resistant *Campylobacter* isolates from diarrheal patients and poultry meat in Shanghai, China.

## Introduction

*Campylobacter jejuni* and *Campylobacter coli* are major causes of bacterial gastroenteritis in the world (Silva et al., 2011). Humans most often become infected by ingesting contaminated food, especially undercooked poultry. For example, a national-scale high-throughput molecular typing study in Scotland had demostrated that chickens were the dominant source of campylobacteriosis(Sheppard et al., 2009). And molecular epidemiology study of *C. coli* in New Zealand suggested poultry as a main source to the burden of human disease (Nohra et al., 2016).

Effective antimicrobial therapy is critical in the treatment of prolonged and severe campylobacteriosis (Silva et al., 2011). The most common antimicrobial agents used in clinic are erythromycin and ciprofloxacin. Tetracyclines have been suggested as an alternative choice but would rarely be used. Intravenous aminoglycosides are also considered in the treatment of serious bacteraemia and other systemic infections due to *Campylobacter* infection (Alfredson and Korolik, 2007). However, the emergence of antimicrobial resistance to a few common clinical drugs have been repeatedly reported in China (Zhang et al., 2010; Ma et al., 2017) and many other countries (Ge et al., 2013; Narvaez-Bravo et al., 2017). Significantly, multidrug resistant clones are emerging in East Asia in recent years. A study on tracking *Campylobacter* contamination along a broiler chicken production chain in China suggested 71.7% of *C. jejuni* and 98.0% of *C. coli* exhibited multidrug resistance (Ma et al., 2014). And 57.1% of *C. jejuni* and 70.9% of *C. coli* from retail chicken and duck were resistant to at least four antibiotics in South Korea (Wei et al., 2016). Multidrug resistance was detected in 99.0% of *C. coli* from diarrheal patients and food-producing animals (Zhang et al., 2014). Our previous study had identified several clones of multidrug resistant *Campylobacter* from infants younger than 2 years of age (Pan et al., 2015).

Several mechanisms responsible for resistance have been described, for example, fluoroquinolone resistance mainly due to mutations in the DNA gyrase gene (*gyrA* gene). Resistance to tetracyclines is conferred by the *tet(O)* gene. Macrolide resistance is the result of modification of the ribosome target binding site by mutation of the 23S rRNA, and activation of the CmeABC multidrug efflux pump leads to resistance to several antimicrobials (Luangtongkum et al., 2009; Iovine, 2013; Wieczorek and Osek, 2013). Interestingly, presences of antimicrobial resistant genes of Gram-positive origin, such as *ermB*, *aadE* and *aadE-sat4-aphA* are also associated with multidrug resistance in *Campylobacter* (Wang et al., 2014).

Antimicrobial resistance of some common clinical drugs is of particular concern. The objectives of the present study were to determine the genetic prerequisites for antibiotic resistance of multidrug resistant *Campylobacter* isolates from diarrheal patients and poultry meat in Shanghai, China.

## Materials and Methods

### Bacterial Collection and Growth Conditions

A total of 548 isolates of *Campylobacter* were collected in Shanghai, China from 2009 to 2014, including 443 isolates from patients with acute watery diarrhea, 97 from chicken meat, 7 from duck meat and 1 from pigeon meat. Speciation of the *Campylobacter* isolates was determined by performing multiple PCR method using genes *16S rRNA* (857 bp for *Campylobacter* genus, upper primer 5′ ATC TAA TGG CTT AAC CAT TAA AC 3′ and lower primer 5′ GGA CGG TAA CTA GTT TAG TAT T 3′), *mapA* (589 bp for *jejuni* species, upper primer 5′ CTA TTT TAT TTT TGA GTG CTT GTG 3′ and lower primer 5′ GCT TTA TTT GCC ATT TGT TTT ATT A 3′) and *ceuE* (462 bp for *coli* species, upper primer 5′ AAT TGA AAA TTG CTC CAA CTA TG 3′ and lower primer 5′ TGA TTT TAT TAT TTG TAG CAG CG 3′) (Denis et al., 1999). *C. jejuni* ATCC 33560 and *C. coli* ATCC 33559 were used as controls in antimicrobial susceptibility testing. The bacteria were incubated on Mueller-Hinton (MH) agar (Hopebiol, Qingdao, China) containing 5% (vol/vol) sheep blood at 42°C under a microaerophilic atmosphere (85% N_2_, 5% O_2_ and 10% CO_2_).

### Antimicrobial Susceptibility Testing

Minimum inhibitory concentrations (MICs) were determined using agar dilution method described by the National Antimicrobial Resistance Monitoring System (NARMS) report (CDC, 2012). Eight antimicrobials belonging to seven classes were selected for antimicrobial susceptibility testing based on their antimicrobial mechanisms and importance for human campylobacteriosis treatment. They included β-lactams (ampicillin, AMP), aminoglycosides (gentamicin, GEN), quinolones (nalidixic acid, NAL and ciprofloxacin, CIP), macrolides (erythromycin, ERY), lincosamides (clindamycin, CLI), tetracyclines (tetracycline, TET) and phenicols (chloramphenicol, CHL), MIC breakpoints of resistance were ≥32 μg/ml for CHL, ≥4 μg/ml for CIP, ≥8 μg/ml for CLI, ≥32 μg/ml for ERY, ≥4 μg/ml for GEN, ≥64 μg/ml for NAL, ≥16 μg/ml for TET. MIC breakpoint for AMP (≥32 μg/ml) was utilized according to MIC interpretive standards for Enterobacteriaceae (CLSI, 2012). The antimicrobials used in this study were purchased from Sigma (St. Louis, MO, United States). *C. jejuni* ATCC 33560 and *C. coli* ATCC 33559 were included in each batch of agar dilution tests for quality control. Multidrug resistance was defined as resistance to three or more antimicrobial groups.

### Pulsed-Field Gel Electrophoresis (PFGE)

Pulsed-field gel electrophoresis was performed on the multidrug-resistant *Campylobacter* identified in this study as previously described (Ribot et al., 2001). Briefly, plugs were prepared using cells removed from the surface of the culture plates. After lysis for 0.5 h, the plugs were washed four times at 54°C in a shaking water bath. A 2-mm-wide slice from each plug was cut and transferred to a tube containing restriction buffer solution (TaKaRa, Dalian, China). The plug slices were incubated in this restriction buffer at room temperature (23–25°C) for 5 min. Genomic DNA was digested with 40 U of *SmaI* (TaKaRa, Dalian, China) at room temperature for 2 h. Restriction fragments were separated by electrophoresis in 0.5 × Tris-Borate-EDTA buffer at 14°C for 18 h using a Chef Mapper electrophoresis system (Bio-Rad, Hercules, CA, United States) with pulse times of 6.75–38.35 s). Gel images were scanned and analyzed using BioNumerics v.6.6 (Applied Maths, Kortrijk, Belgium). Dendrograms were created from a matrix of band matching using the Jaccard coefficient and the unweighted pair group method with arithmetic mean analysis (UPGMA).

### PCR Identification of the Antibiotic Resistant Genes

Putative antibiotic resistant genes *gyrA*, 23s rRNA, *tet (O)*, *aadE*, *ermB*, *aadE-sat4-aphA* and the *cmeR-cmeABC* intergenic region were amplified and sequenced using primers listed in **Table 2**. Templates of the 151 multidrug resistant *Campylobacter* isolates and the negative control (*Escherichia coli* DH5α) were prepared by a boiling method. Briefly, an overnight broth culture (0.5 ml) was heated at 100°C for 10 min and centrifuged at 12,000 *g* for 5 min. The supernatant was used as DNA template. The cycling program was denaturation at 94°C for 1 min, annealing at a temperature specific to each primer pair for 1 min, and extension at 72°C for 2 min. The sequences were assembled using Laser-gene Seqman II software (DNAStar, Inc., Madison, WI, United States), and aligned using the CLUSTALWroutine of MegAlign software (v.6.1, DNA Star, Inc.). All PCR reactions were performed in duplicate.

### Nucleotide Sequence Accession Numbers

The sequences of the *cmeR-cmeABC* intergenic regions have been deposited in GenBank, and their accession numbers were listed in Supplementary Table 1.

### Ethics Statement

The surveillance and sampling protocols were approved by Shanghai Center for Disease Control and Prevention Review Board. This study did not involve any health-related patient interventions. A verbal informed consent was obtained from each subject; however, the patient identities have not been disclosed at any stage. The institutional board was informed of the specific needs with reference to the study setting and approved this mode of consent. A check box was included in the data form to document the consent-taking procedure.

## Results

### Identification of Multidrug-Resistant *Campylobacter* Isolates

The 548 *Campylobacter* isolates were identified as 372 *C. jejuni* and 176 *C. coli*. **Table 1** provides details on the origins and sampling dates for all isolates. Among them, a total of 151 *Campylobacter* isolates (32 *C. jejuni* and 119 *C. coli*) were resistant to three or more antimicrobials, including 99 from diarrheal patients and 52 from poultry meat. Multidrug-resistance rates were much higher in *C. coli* than in *C. jejuni* (67.6%, *n* = 119 versus 8.6%, *n* = 32, respectively) (**Table 2**). A large proportion of the 151 isolates were resistant to tetracycline (99.3%), ciprofloxacin (97.4%), erythromycin (80.1%), gentamicin (76.2%) and clindamycin (71.5%). And the antimicrobial resistance rates in *C. jejuni* and *C. coli* were 100, 100, 65.6, 53.1, 37.5% and 99.2, 96.6, 84, 82.3, 80.7%, respectively (**Table 3**).

**Table 1:** Sources of the 548 *Campylobacter* isolates from 2009 to 2014.

Isolated year	Sources (No.)							
	Human (*n* = 443)		Chicken meat (*n* = 97)		Duck meat (*n* = 7)		Pigeon meat (*n* = 1)	
	*C. jejuni*	*C. coli*	*C. jejuni*	*C. coli*	*C. jejuni*	*C. coli*	*C. jejuni*	*C. coli*
2009	3	1	0	12	0	0	0	0
2010	24	3	6	2	0	0	0	0
2011	137	51	12	6	1	0	0	1
2012	53	21	5	8	0	0	0	0
2013	39	24	0	0	0	0	0	0
2014	60	27	26	20	6	0	0	0
Total	316	127	49	48	7	0	0	1

**Table 2:** Primers for the identification of antimicrobial resistance genes of the multidrug resistance *Campylobacter jejuni* and *Campylobacter coli.*

Antimicrobial resistant gene	Primers	Primer sequences	Amplified fragment length (bp)	Reference
*gyrA*	QRDR- for	GAGYGTTATTATMGGTCGTGC	286	Barco et al., 2010
	QRDR- rev	TTCAGTATAACGCATYGCAGC		
*erm (B)*	erm (B)- for	GGGCATTTAACGACGAAACTGG	738, 753 or 765	Wang et al., 2014
	erm (B)- rev	CTGTGGTATGGCGGGTAAGT		
23s rRNA	23s rRNA- for	TTAGCTAATGTTGCCCGTACCG	697	Alonso et al., 2005
	23s rRNA- rev	AGCCAACCTTTGTAAGCCTCCG		
*tet (O)*	tet (O)- for	AGTTTCTGCAAAGGATGGCAT	447	Qin et al., 2011
	tet (O)- rev	GATTGACCTTCAGGCGTTGAT		
*aadE*	aadE-for	GCTGCCGCTGGAACT	527	Wang et al., 2014
	aadE-rev	TCTTTTGCCGAATCACA		
*aadE-sat4-aphA*	aadE-sat4-aphA-for	AAAGGATTGTGCCGTAA	1628	
	aadE-sat4-aphA- rev	TGCTGTCTCCCAGGTC		
*cmeR/cmeA* (*C. jejuni*)	CmeF1	TTGCACTATGTTAAAAGAACC	396	Perez-Boto et al., 2010
	CmeR2	TGTCCGTAAGCCGAAT		
*cmeR/cmeA* (*C. coli*)	CmecoliF3	AATGTTTTAGCCGATACT		
	CmecoliR4	AACACCGCTTACTTGAGG		

**Table 3:** The 151 multidrug-resistant *Campylobacter* isolates and the presence/mutant of the associated antibiotic resistant genes.

Species (No.)	No. (%) of the antibiotic resistant isolates					No. (%) of the isolates that carry antibiotic resistant genes					
	CIP	TET	GEN	ERY	CLI	*gyrA at codon* 86	*ermB*	23s rRNA A2075G	*tet (O)*	*aadE*	*aadE-sat4-aphA*
*C. jejuni* (*n* = 32)	32 (100)	32 (100)	17 (53.1)	21 (65.6)	12 (37.5)	31 (96.9)	1 (3.1)	1 (3.1)	31 (96.9)	3 (9.4)	1 (3.1)
*C. coli* (*n* = 119)	115 (96.6)	118 (99.2)	98 (82.3)	100 (84)	96 (80.7)	119 (100)	30 (25.2)	55 (46.2)	117 (98.3)	86 (72.2)	9 (7.6)
Total (*n* = 151)	147 (97.4)	150 (99.3)	115 (76.2)	121 (80.1)	108 (71.5)	150 (99.3)	31 (20.5)	56 (37.1)	148 (98)	89 (58.9)	10 (6.6)

### Distribution and Genetic Patterns of the Multidrug-Resistant *Campylobacter* Isolates

**FIGURE 1:**
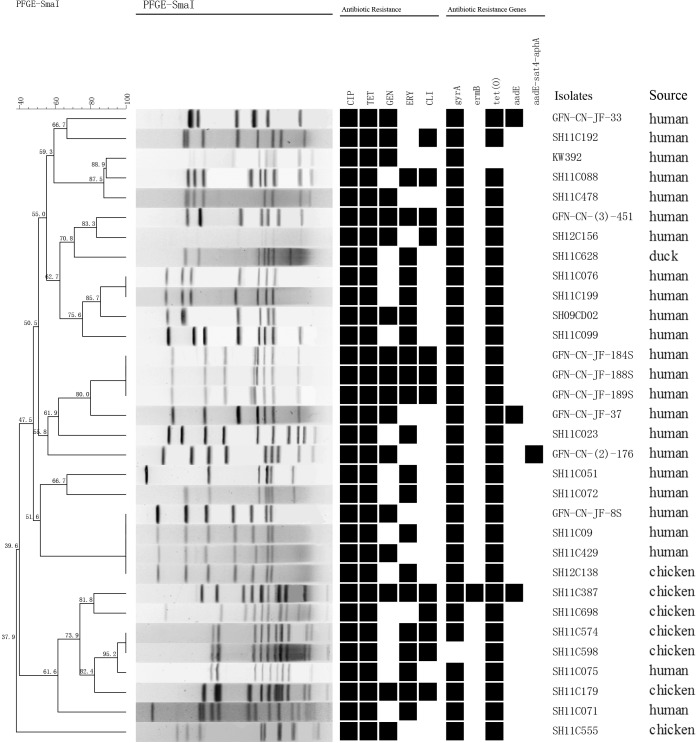
Dendrogram of PFGE profiles among the 32 multidrug-resistant *Campylobacter jejuni*. PFGE assay resulted into 26 different patterns. Corresponding antibiotic resistant patterns, antimicrobial resistant genes or mutants were listed for each isolate. CIP, ciprofloxacin; TET, tetracycline; GEN, gentamicin; ERY, erythromycin; CLI, clindamycin.

**FIGURE 2:**
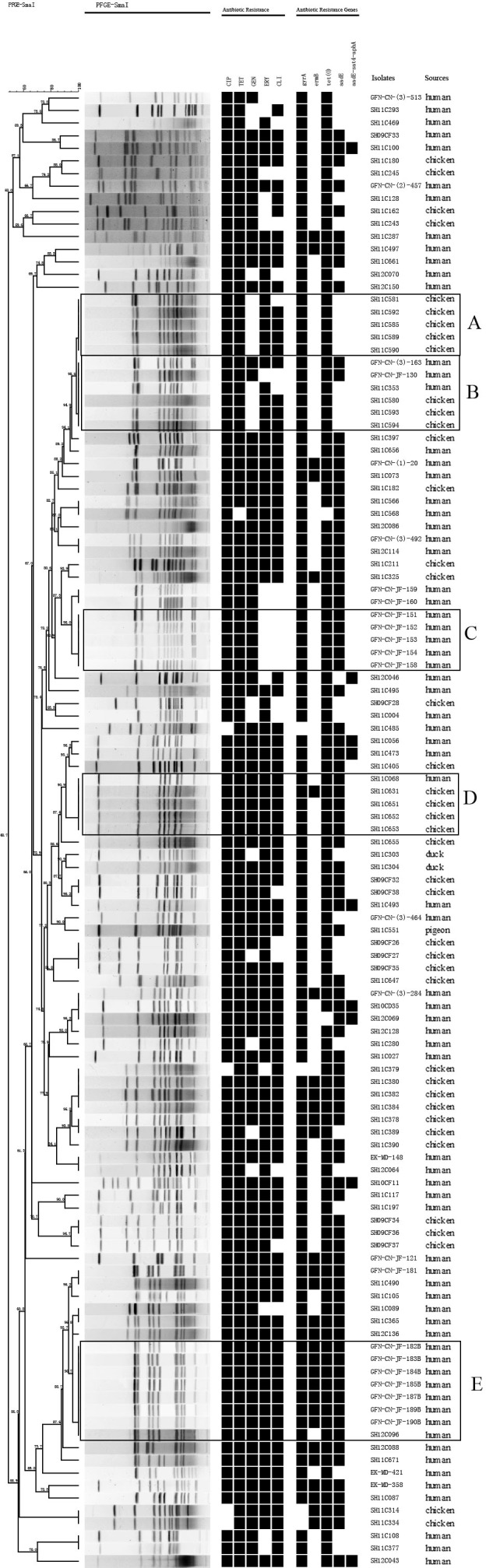
Dendrogram of PFGE profiles among the 119 multidrug-resistant *Campylobacter coli*. PFGE assay resulted into 77 different patterns. Corresponding antibiotic resistant patterns, antimicrobial resistant genes or mutants were listed for each isolate. Five prevalent genotypes (A–E) were identified. CIP, ciprofloxacin; TET, tetracycline; GEN, gentamicin; ERY, erythromycin; CLI, clindamycin.

Genetic relatedness of the 151 multidrug-resistant *Campylobacter* isolates was analyzed by PFGE. Genetic diversity was significant among the 32 *C. jejuni* isolates, with 26 distinct PFGE genotypes (**Figure 1**). Interestingly, some human associated isolates shared identical PFGE genotype with those from chicken. For example, *C. jejuni* GFN-CN-JF-8S, SH11C09, SH11C429 isolated from patients were grouped together with SH12C138, which was isolated from chicken (**Figure 1**). In contrast, the 119 *C. coli* isolates represented 77 genotypes. It was also observed that isolates from different sources were grouped together. For example, in genotype B, *C. coli* SH11C353, GFN-CN-(3)-163 and GFN-CN-JF-130 that were isolated from human had identical genotype with the other three from chicken. And in genotype D, *C. coli* SH11C068 from human and the other 4 from chicken were grouped together (**Figure 2**).

### Identification of Antibiotic Resistant Genes

Among the 151 multidrug-resistant *Campylobacter* isolates, the numbers of isolates carried *tet (O)*, *aadE, ermB*, and *aadE-sat4-aphA* were 148 (98%), 89 (58.9%), 31 (20.5%), and 10 (6.6%), respectively. Almost all (*n* = 150, 99.3%) had *gyrA* mutation at codon 86 that causes ciprofloxacin resistance. And the 23s rRNA A2075G point mutation, which is regarded to be associated with erythromycin resistance, was found in 56 (37.1%) isolates (**Table 3**). Isolates belonging to the same genotypes usually carry the same antimicrobial resistance genes and therefore exhibited similar antimicrobial resistance patterns. However, some exceptions were also observed. For example, *C. coli* SH11C631 carried a putative antibiotic resistance associated gene *ermB*, and GFN-CN-(3)-163 and GFN-CN-JF-130 carried *aadE*. We analyzed the relationship between PFGE genotype and the presence of antimicrobial resistance genes of Gram-positive origin *ermB*, *aadE* and *aadE-sat4-aphA*. No obvious links can be found.

### Genetic Polymorphisms of the *cmeR-cmeABC* Intergenic Regions

**FIGURE 3:**
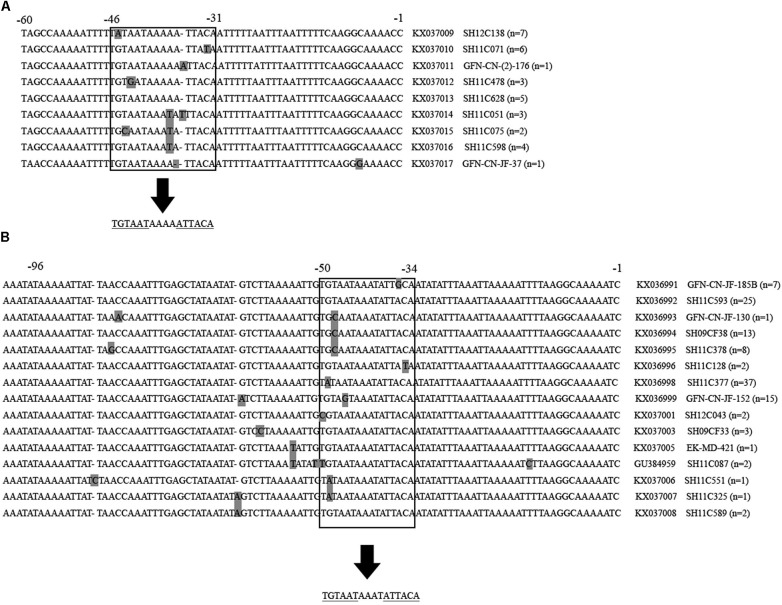
Partial multiple alignment of the intergenic regions between *cmeR* and *cmeABC* identified in multidrug-resistant *Campylobacter jejuni* and *Campylobacter coli* isolates. Polymorphisms are shown in gray squares. The inverted repeat regions are shown inside the open squares, and under the arrows the consensus sequence is shown. Repeated sequences are underlined. Positions are indicated on the top of the alignment. Partial sequences are shown. **(A)** Alignment of the 94 bp intergenic region of *Campylobacter jejuni* isolates. A total of 9 novel identified sequences are presented, followed by the accession number, a representative isolates of each sequence type and the number of isolates belonging to this sequence type. **(B)** Alignment of the 107 bp intergenic region of *Campylobacter coli* isolates. A total of 15 sequences were identified, including 14 novel ones, followed by the accession number, a representative isolates of each sequence type and the number of isolates belonging to this sequence type.

Gene mutations were identified at the inverted repeat regions and flanking regions in the 32 *C. jejuni* and 119 *C. coli* multidrug-resistant isolates. As shown in **Figure 3**, the intergenic region of *C. jejuni* was 94 nucleotides, and the inverted repeat region was located from −46 to −31 upstream of the *cmeABC* (consensus sequence: TGTAATAAAAATTACA). While, the intergenic region of *C. coli* was 107 nucleotides, and the inverted repeat region was located from −50 to −35 upstream of the *cmeABC* (consensus sequence: TGTAATAAATATTACA). Nine and 15 different sequence types were determined in *C. jejuni* and *C. coli* isolates, respectively (**Figure 3**).

## Discussion

Antibiotic resistance is recognized as a global problem in human medicine. Several surveillance programs monitoring antimicrobial resistance of *Campylobacter* to ciprofloxacin, erythromycin, gentamicin, tetracycline, *etc.* have been well established in the developed world, such as the U.S. National Antimicrobial Resistance Monitoring Systems, the European Food Safety Authority, the European Centre for Disease Prevention and Control and national surveillance programs in several European countries (Ge et al., 2013). Compared with that of developed countries, knowledge on the resistance trends of *Campylobacter* in developing countries is more limited, which is largely hindered by the lack of an adequate surveillance system. In clinical settings, tetracyclines, fluoroquinolones and macrolides are widely used as the first choice to treat *Campylobacter* infections because of their low cost and ready availability. In this study, the resistance rates of tetracycline, ciprofloxacin and erythromycin were 99.3, 97.4, and 80.1%, which were much higher than those reported in Canada (Narvaez-Bravo et al., 2017), the United States and European Union (Ge et al., 2013).

As a zoonotic pathogen, *Campylobacter* has a broad animal reservoir. Antibiotic usage in both animal agriculture and human medicine can influence the development of antibiotic resistance (Luangtongkum et al., 2009). Unfortunately, multidrug resistant *Campylobacter* isolates are now creeping from food supply chain (Ma et al., 2014, 2017; Zhang et al., 2014, 2016; Wang et al., 2016; Wei et al., 2016; Zhong et al., 2016) into our community (Pan et al., 2015; Zhang et al., 2017). Multidrug resistant *Campylobacter* isolates are big risks to public health. Establishment of integrated surveillance networks that can monitering their transmission should be taken into consideration in China and other East Asia countries.

Molecular epidemiological studies have contributed to our understanding of transmission of foodborne bacteria. Multilocus sequence typing and PFGE have been developed as robust typing tools for this major pathogen. In this study, PFGE analysis was performed on the 151 multidrug resistant isolates, and a total of 103 PFGE genotypes were determined. Five prevalent genotypes (A–E) were identified in *C. coli*. It is noteworthy that 7 *C. coli* isolates in genotype E carried the same antibiotic resistance genes and resist to all the antibiotics (**Figure 2**). They were isolated from a public hospital in the same period of time (data not shown). In genotype B, isolates from human and chicken were grouped together, indicating this clone might be able to spread across species. We have added these clones into our high-risk clone database. Further studies on the spread of these clones should be carried out in order to rapidly control hospital-acquired infections and identify disease outbreaks. As the world’s third biggest city by population, Shanghai has 26 million people, including over 9 million migrant workers (Statistics of population and family planning, 2016). It is worthy of our concern that floating population may become a vehicle of the transmission of this major pathogen. Therefore, to effectively reduce *Campylobacter* infection in human, control and prevention strategies should be aimed at both food supply chain and susceptible population levels.

Bacterial resistance to a certain antibiotic is usually multifactorial. Some molecular mechanisms have been proven (Luangtongkum et al., 2009). By investigating the molecular mechanisms of multidrug resistant patterns, we found that antibiotic resistance phenotypes of the isolates correlated well with their antibiotic resistance genes. Almost all (150, 99.3%) had *gyrA* mutation at codon 86 that causes ciprofloxacin resistance; *tet (O)*, the tetracycline resistance associated gene, was identified in 148 (98%) isolates; 23s rRNA A2075G point mutation, which is regarded to be associated with erythromycin, was found in 56 (37.1%) isolates; and 89 (58.9%) and 10 (6.6%) isolates carried *aadE* and *aadE-sat4-aphA* cluster respectively, and were regarded associated with both gentamicin and clindamycin resistance. More studies should be carried out on the horizontal spread of these Gram-positive origin genes by genome comparison (Florez-Cuadrado et al., 2017). In addition, the *cmeR-cmeABC* intergenic regions were investigated. Gene mutations were identified at the inverted repeat regions and flanking regions in both *C. jejuni* and *C. coli* isolates, and 9 and 15 differents sequence types were determined, respectively. These sequence types have potentials to be developed as tools for detecting multidrug resistant *Campylobacter*. Isolates belonging to the same genotype usually carry the same antimicrobial resistance genes and therefore exhibited similar antimicrobial resistance patterns. However, we did not find obvious links between PFGE genotype and the presence of antimicrobial resistance genes based on the limited data in this study.

## Conclusion

This study identified 151 multidrug resistant *Campylobacter* isolates, and identified several clones that spread among human and poultry. Molecular assay of the antibiotic resistance genes revealed the mechanism of the antibiotic resistant phenotype. The results of this study may help better understand the situation of antibiotic resistance trends in China, and provide guidelines of antibiotic use for treatment of *Campylobacter* infection in clinic.

## Author Contributions

YD, JZ, and XX contributed to the conception of the study. CW and YY contributed significantly to analysis and manuscript preparation. HP performed data analysis and wrote the manuscript. YuL and AW helped perform analysis with constructive discussions. YoL and XZ collected samples and conducted the experiments.

## Conflict of Interest Statement

The authors declare that the research was conducted in the absence of any commercial or financial relationships that could be construed as a potential conflict of interest.
